# Impaired suppression of fatty acid release by insulin is a strong predictor of reduced whole‐body insulin‐mediated glucose uptake and skeletal muscle insulin receptor activation

**DOI:** 10.1111/apha.14249

**Published:** 2024-11-01

**Authors:** Michael W. Schleh, Benjamin J. Ryan, Cheehoon Ahn, Alison C. Ludzki, Douglas W. Van Pelt, Lisa M. Pitchford, Olivia K. Chugh, Austin T. Luker, Kathryn E. Luker, Dmitri Samovski, Nada A. Abumrad, Charles F. Burant, Jeffrey F. Horowitz

**Affiliations:** ^1^ Substrate Metabolism Laboratory, School of Kinesiology University of Michigan Ann Arbor Michigan USA; ^2^ Center for Molecular Imaging, Department of Radiology University of Michigan Ann Arbor Michigan USA; ^3^ Biointerfaces Institute University of Michigan Ann Arbor Michigan USA; ^4^ Department of Medicine Washington University School of Medicine St. Louis Missouri USA; ^5^ Division of Metabolism, Endocrinology, and Diabetes, Department of Internal Medicine University of Michigan Ann Arbor Michigan USA

**Keywords:** CD36, insulin resistance, lipid droplet, obesity, skeletal muscle

## Abstract

**Aim:**

To examine factors underlying why most, but not all, adults with obesity exhibit impaired insulin‐mediated glucose uptake, we compared: (1) adipose tissue fatty acid (FA) release, (2) skeletal muscle lipid droplet (LD) characteristics, and (3) insulin signalling events, in skeletal muscle of adults with obesity with relatively high versus low insulin‐mediated glucose uptake.

**Methods:**

Seventeen adults with obesity (BMI: 36 ± 3 kg/m^2^) completed a 2 h hyperinsulinemic–euglycemic clamp with stable isotope tracer infusions to measure glucose rate of disappearance (glucose Rd) and FA rate of appearance (FA Ra). Skeletal muscle biopsies were collected at baseline and 30 min into the insulin infusion. Participants were stratified into HIGH (*n* = 7) and LOW (*n* = 10) insulin sensitivity cohorts by their glucose Rd during the hyperinsulinemic clamp (LOW< 400; HIGH >550 nmol/kgFFM/min/[μU/mL]).

**Results:**

Insulin‐mediated suppression of FA Ra was lower in LOW compared with HIGH (*p* < 0.01). In skeletal muscle, total intramyocellular lipid content did not differ between cohorts. However, the size of LDs in the subsarcolemmal region (SS) of type II muscle fibres was larger in LOW compared with HIGH (*p* = 0.01). Additionally, insulin receptor‐β (IRβ) interactions with regulatory proteins CD36 and Fyn were lower in LOW versus HIGH (*p* < 0.01), which aligned with attenuated insulin‐mediated Tyr phosphorylation of IRβ and downstream insulin‐signalling proteins in LOW.

**Conclusion:**

Collectively, reduced ability for insulin to suppress FA mobilization, with accompanying modifications in intramyocellular LD size and distribution, and diminished IRβ interaction with key regulatory proteins may be key contributors to impaired insulin‐mediated glucose uptake commonly found in adults with obesity.

## 1 INTRODUCTION

Obesity has now surpassed 40% of the US population,^1^ and is a major risk factor for the development of chronic metabolic conditions such as insulin resistance,^2^ which in turn underlies many obesity‐related diseases.^3^ Although most adults with obesity are insulin resistant for glucose uptake, some remain relatively insulin sensitive,^4^, ^5^ and factors helping to protect against the development of insulin resistance in these individuals are not clear. Work from our laboratory^6^, ^7^ and others^8^, ^9^, ^10^ demonstrated the rate of fatty acid (FA) release/mobilization into the systemic circulation is directly linked with whole‐body insulin sensitivity among adults with obesity. The vast majority of FA in the systemic circulation are derived from abdominal subcutaneous adipose tissue (aSAT),^11^ and we contend the variability in the regulation of FA release from aSAT is an important contributor to the differences in the magnitude of insulin resistance observed among adults with obesity.

The link between excessive systemic FA mobilization and whole‐body insulin resistance has been largely attributed to high rates of FA uptake into the intramyocellular region of skeletal muscle and the resultant over‐accumulation of bioactive lipids.^12^, ^13^, ^14^, ^15^ In particular, some bioactive lipids reported to promote insulin resistance include diacylglycerol (DAG) localized to the muscle membrane,^16^ total ceramide^17^, ^18^ and long‐chain fatty acyl‐CoA.^19^, ^20^ Specifically, the over‐accumulation of the bioactive lipids DAG and ceramides are proposed to activate PKC family proteins (PKC ε, θ, ζ), resulting in Ser/Thr phosphorylation of insulin receptor substrate‐1 (IRS‐1), and reduced phosphorylation of AKT.^21^, ^22^, ^23^ However, the contribution of each of these lipid intermediates to insulin resistance has been challenged,^24^, ^25^, ^26^ and PKC‐independent mechanisms may also underlie poor activation in the development of insulin resistance. Intramyocellular lipids are primarily stored within lipid droplets (LDs) composed of a triacylglycerol‐rich core surrounded by a phospholipid monolayer containing proteins that regulate FA kinetics and direct intracellular LD trafficking.^27^, ^28^ Importantly, the metabolic impact of LDs can vary depending on their location within the myocyte. For example, LDs within the intramyofibrillar region (IMF; toward the centre of the myocyte) are proposed to support mitochondrial respiration to help meet energy requirements during muscle contraction, and LDs in the subsarcolemmal region (SS; toward the periphery of the myocyte) primarily support the metabolic demand from the plasma membrane.^29^, ^30^ The intracellular distribution of LDs within the myocyte has also been linked with insulin resistance, where LD accumulation in the SS region has been associated with insulin resistance and type 2 diabetes.^31^, ^32^, ^33^ It is possible that impaired insulin sensitivity due to the accumulation of SS LDs may result in increased release of lipid intermediates near the cell membrane, where insulin signalling is initiated. Differences in the number and size of intramyocellular LDs within the IMF and SS regions may also impact insulin sensitivity, but these relationships are not firmly established. One principal aim of this study was to compare the number and size of LDs within the IMF and SS regions of type I and type II skeletal muscle fibres from a cohort of adults with HIGH versus LOW insulin sensitivity.

The disruption of insulin signalling induced by the intramyocellular lipids have classically been attributed to modifications in the phosphorylation of signalling proteins downstream of the insulin receptor (IRβ) (e.g., IRS‐1, phosphoinositide 3‐kinase (PI3K), and AKT). However, other reports suggest lipid‐induced disruption directly at IRβ may also contribute to impaired glucose uptake.^34^ The membrane glycoprotein, cluster of differentiation 36 (CD36) is not only well known for its role in regulating long‐chain FA transport,^35^, ^36^, ^37^ but also has been implicated in functions related to cellular metabolism and IRβ phosphorylation,^34^, ^38^ thereby modifying downstream signalling.^39^ The physical interaction between CD36 and IRβ has been found to increase Tyr phosphorylation of IRβ via CD36‐mediated recruitment of the Src‐family tyrosine kinase, Fyn.^34^ Conversely, previous reports demonstrated elevated saturated FA availability attenuated Fyn recruitment to the CD36‐IRβ complex,^34^, ^39^, ^40^ leading to blunted insulin signalling.^34^, ^39^ These previous experiments were conducted in vitro, and whether differences in systemic FA availability among adults with obesity may contribute to modifications in CD36 recruitment of Fyn to IRβ and modify IRβ phosphorylation and downstream insulin action in human skeletal muscle is not known. The second major aim of this study was to compare basal‐ and insulin‐mediated interaction between CD36 and Fyn with IRβ, as well as insulin‐stimulated signalling events in skeletal muscle from a cohort of adults with high versus low insulin sensitivity.

## 2 RESULTS

### 2.1 Cohort stratification based on insulin‐mediated glucose uptake

**FIGURE 1:**
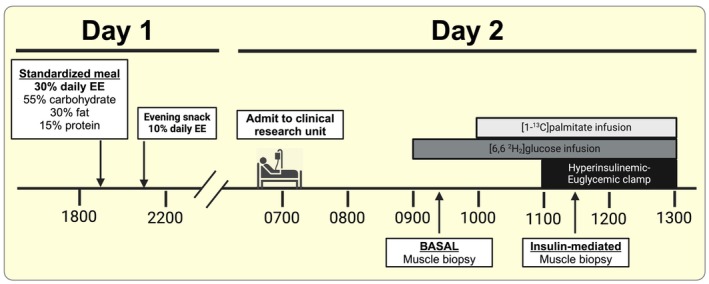
Clinical trial timeline of sample collection. Insulin‐mediated skeletal muscle biopsies were collected 30 min into the hyperinsulinemic clamp. EE, energy expenditure. Created with BioRender.com.

**FIGURE 2:**
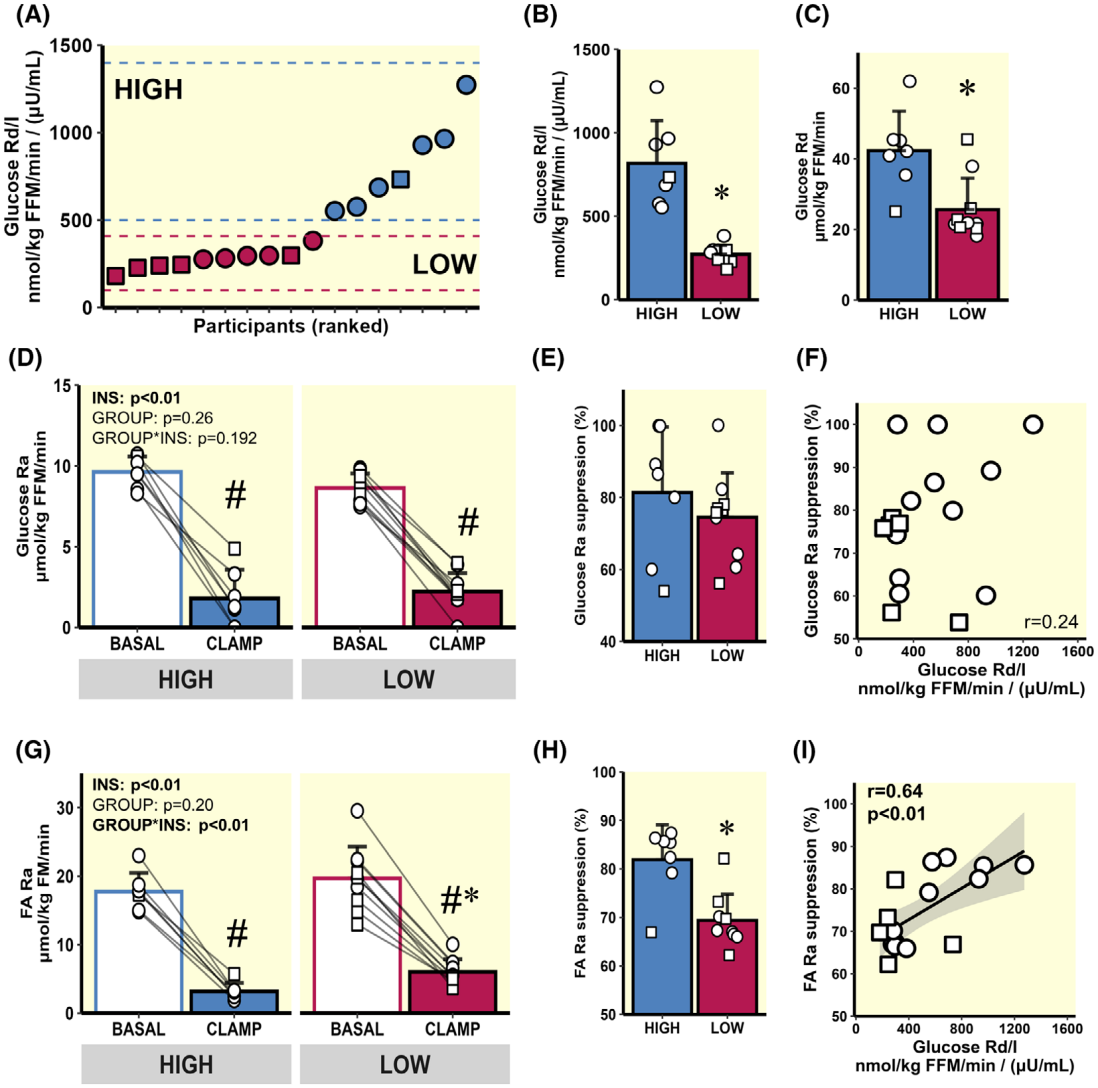
Insulin sensitivity variability across all participants and substrate kinetics. (A) Glucose Rd/I variability across all participants. (B and C) Stratification into HIGH (*n* = 7) and LOW (*n* = 10) cohorts—expressed as glucose Rd normalized to insulin [Glucose Rd/I: nmol/kg FFM/min/(μU/mL)], (B), and glucose Rd normalized only to lean body mass [(glucose Rd: μmol/kg FFM/min], (C). (D) Basal and insulin‐mediated glucose Ra. (E) Glucose Ra % suppression. (F) Correlation between glucose Rd/I and glucose Ra suppression from all participants. (G) Basal and insulin‐mediated FA Ra. (H) FA Ra % suppression. (I) Correlation between glucose Rd/I and FA Ra suppression from all participants. ○ = Female, □ = Male. ^#^ *p* < 0.05 main‐effect for insulin (basal vs. clamp). *p < 0.05 LOW versus HIGH. Data are expressed as mean ± SD.

Table 1 presents the characteristics of the men (*n* = 6) and women (*n* = 11) with obesity (BMI = 30–40 kg/m^2^) who participated in the series of metabolic assessments conducted in this study (Figure 1). As anticipated, rates of insulin‐mediated glucose uptake (measured as glucose Rd/I during the insulin clamp) varied widely among participants. Glucose Rd/I ranged from 181 to 382 nmol/kg FFM/min/(μU/mL) for participants in the LOW cohort and from 552 to 1274 nmol/kg FFM/min/(μU/mL) for participants in the HIGH cohort (Figure 2A,B). The striking differences in glucose Rd/I were present between LOW and HIGH cohorts regardless of whether glucose Rd was normalized to insulin concentration during the clamp (Figure 2B,C).

**TABLE 1:** Participant characteristics.

	HIGH (*n* = 7)	LOW (*n* = 10)	*p*‐value
Sex (M/F)	1/6	5/5	
Age (years)	32 ± 9	31 ± 8	0.75
Body mass (kg)	93 ± 9	105 ± 11*	0.02
BMI (kg/m^2^)	35 ± 3	36 ± 3	0.18
Fat mass (kg)	43 ± 5	46 ± 5	0.22
Fat free mass (kg)	50 ± 10	60 ± 9	0.06
Body fat %	46 ± 6	43 ± 5	0.22
Liver fat (%)	3.4 ± 2.0	9.5 ± 8.3	0.19
Visceral fat (cm^2^)	107 ± 49	169 ± 58	0.10
Plasma parameters			
Fasting plasma glucose (mM)	4.6 ± 0.4	4.7 ± 0.4	0.71
Fasting plasma insulin (μU/mL)	6.3 ± 2.5	16.8 ± 4.7*	3.4e^−05^
HOMA‐IR	1.3 ± 0.6	3.5 ± 1.0*	6.3e^−05^
HbA1c (%)	5.3 ± 0.4	5.4 ± 0.2	0.73
Plasma FA (μM)	438 ± 197	544 ± 176	0.27
Plasma triacylglycerol (mg/dL)	58 ± 34	80 ± 38	0.24
Plasma HDL (mg/dL)	44 ± 10	38 ± 12	0.27
Plasma cholesterol (mg/dL)	141 ± 15	143 ± 32	0.88
Plasma CRP (mg/L)	11 ± 14	9 ± 11	0.79
Plasma IL6 (ρg/mL)	3.0 ± 1.4	3.3 ± 2.2	0.74
Plasma HMW adiponectin (ng/mL)	2581 ± 1412	1658 ± 1261	0.09
Plasma adiponectin (total) (ng/mL)	5978 ± 2139	3840 ± 2017	0.06
Plasma leptin (ng/mL)	61 ± 29	51 ± 19	0.44

*Note*: Data are mean ± SD.

Abbreviations: BMI, body mass index; CRP, C‐reactive protein; FA, fatty acid; HDL, high‐density lipoprotein; HIGH, high insulin sensitivity; HMW adiponectin, high‐molecular weight adiponectin; HOMA‐IR, homeostatic model for insulin resistance; LOW, low insulin sensitivity.

* *p* < 0.05 LOW versus HIGH.

### 2.2 Insulin effects on hepatic glucose production and FA release from adipose tissue

As expected, there was a large reduction in hepatic glucose production (glucose Ra) in response to insulin during the clamp (Figure 2D). Interestingly, despite the robust difference in insulin‐mediated glucose Rd/I between LOW and HIGH, the suppression in glucose Ra (index of hepatic insulin sensitivity) was similar between groups (Figure 2E) and did not correlate with Rd/I (Figure 2F). In contrast, insulin‐mediated FA Ra suppression was greater in HIGH compared with LOW (Figure 2G,H). Additionally, insulin‐mediated FA Ra suppression was positively correlated with glucose Rd/I across all study participants (Figure 2I; r = 0.64; *p* < 0.01), suggesting a reduced ability to suppress FA mobilization from adipose tissue in response to insulin may predict an impairment in whole‐body insulin‐mediated glucose uptake.

### 2.3 Intramyocellular LD storage in type I and II skeletal muscle fibres

**FIGURE 3:**
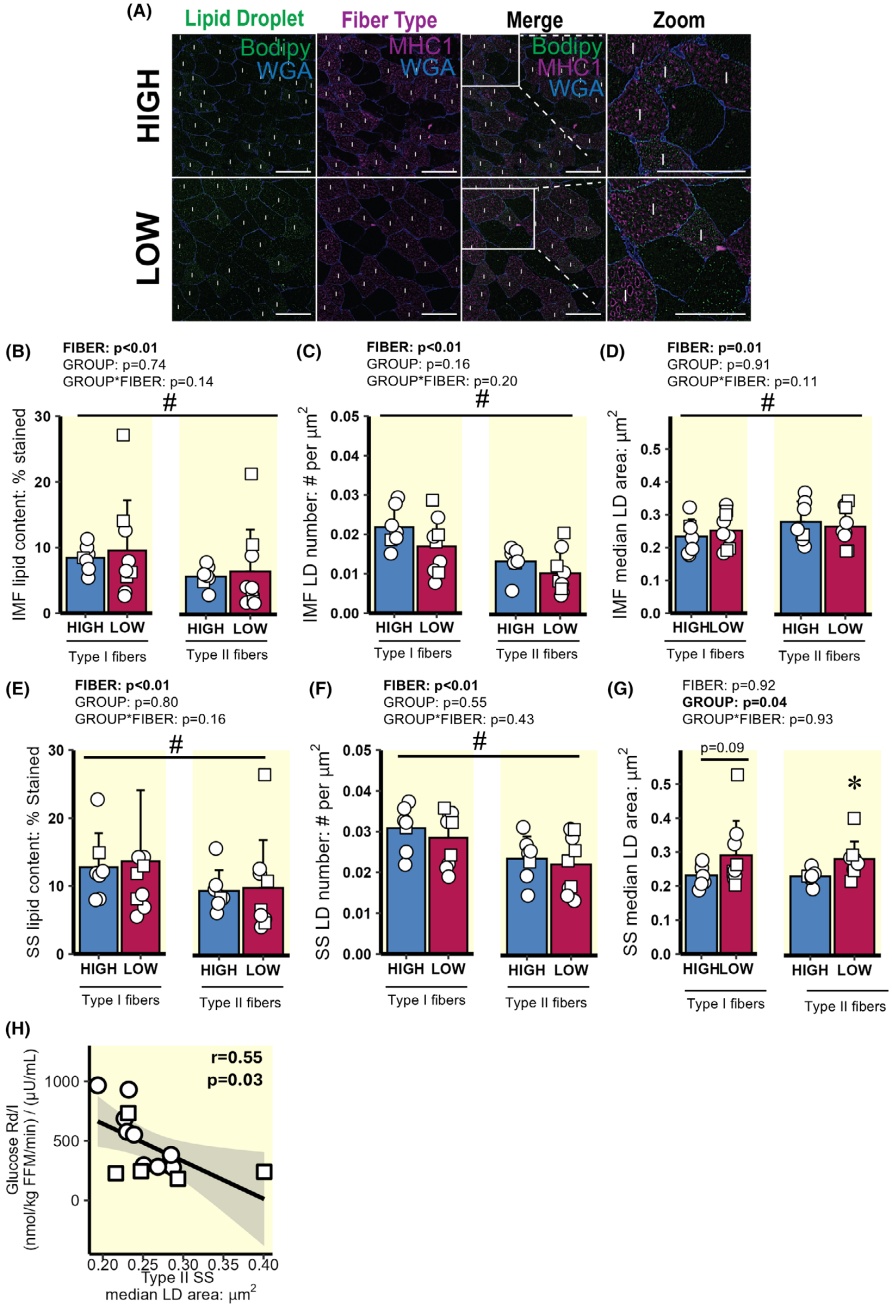
Intramyocellular LD characteristics and distribution in type I and II skeletal muscle fibres. (A) Representative image in skeletal muscle of type I and II fibres from HIGH and LOW. Image contains bodipy stain (LD, green), myofibre stain positive for MHC I (type I fibres, purple), and wheat germ agglutinin (WGA, membranes, blue). The region inside the white boxes is enlarged for a clearer view. Magnification, x20. Scale bar, 100 μm for all images. “I” denotes type I muscle fibre. (B–G) Lipid content and LD characteristics within the IMF and SS region in type I and type II muscle fibres. (B) IMF lipid content (represented as % stained). (C) IMF LD number (# LDs per μm^2^). (D) IMF median LD area per fibre (μm^2^). (E) SS lipid content. (F) SS LD number. (G) SS LD area per muscle fibre. (H) Correlation between glucose Rd/I and SS LD area in type II fibres. ○ = Female, □ = Male; *n* = 6 in HIGH and *n* = 10 in LOW. ^#^ *p* < 0.05 main effect for fibre type (type I vs. type II muscle fibres). *p < 0.05 main effect for group (LOW vs. HIGH), with post‐hoc analysis identifying significant difference for HIGH versus LOW. Data are expressed as mean ± SD.

Total skeletal muscle lipid content was not different between HIGH and LOW (7.8 ± 2.0 vs. 8.5 ± 2.9% stained, respectively; *p* = 0.6). As anticipated, total lipid content was greater in type I versus type II muscle fibres, and this was the case for both cohorts (type I = 9.3 ± 4.9 vs. type II = 6.2 ± 4.1% stained; *p* < 0.01; Figure 3A). Neither total lipid content within the IMF and SS regions of muscle fibres (Figure 3B,E, respectively) nor the number of LDs per μm^2^ in the IMF and SS regions (Figure 3C,F, respectively) were different between HIGH versus LOW groups in either fibre type. However, LD size (measured by median LD area in each muscle fibre) within the SS region was larger in LOW versus HIGH (Figure 3G); this difference in LD area between groups was statistically significant in type II fibres (*p* = 0.01), with a trend for a greater LD area in LOW versus HIGH in type I fibres (*p* = 0.09). Interestingly, LD area in type II fibres was negatively correlated with insulin‐mediated glucose uptake (r = −0.52, *p* = 0.03; Figure 3H).

### 2.4 Skeletal muscle insulin signalling

**FIGURE 4:**
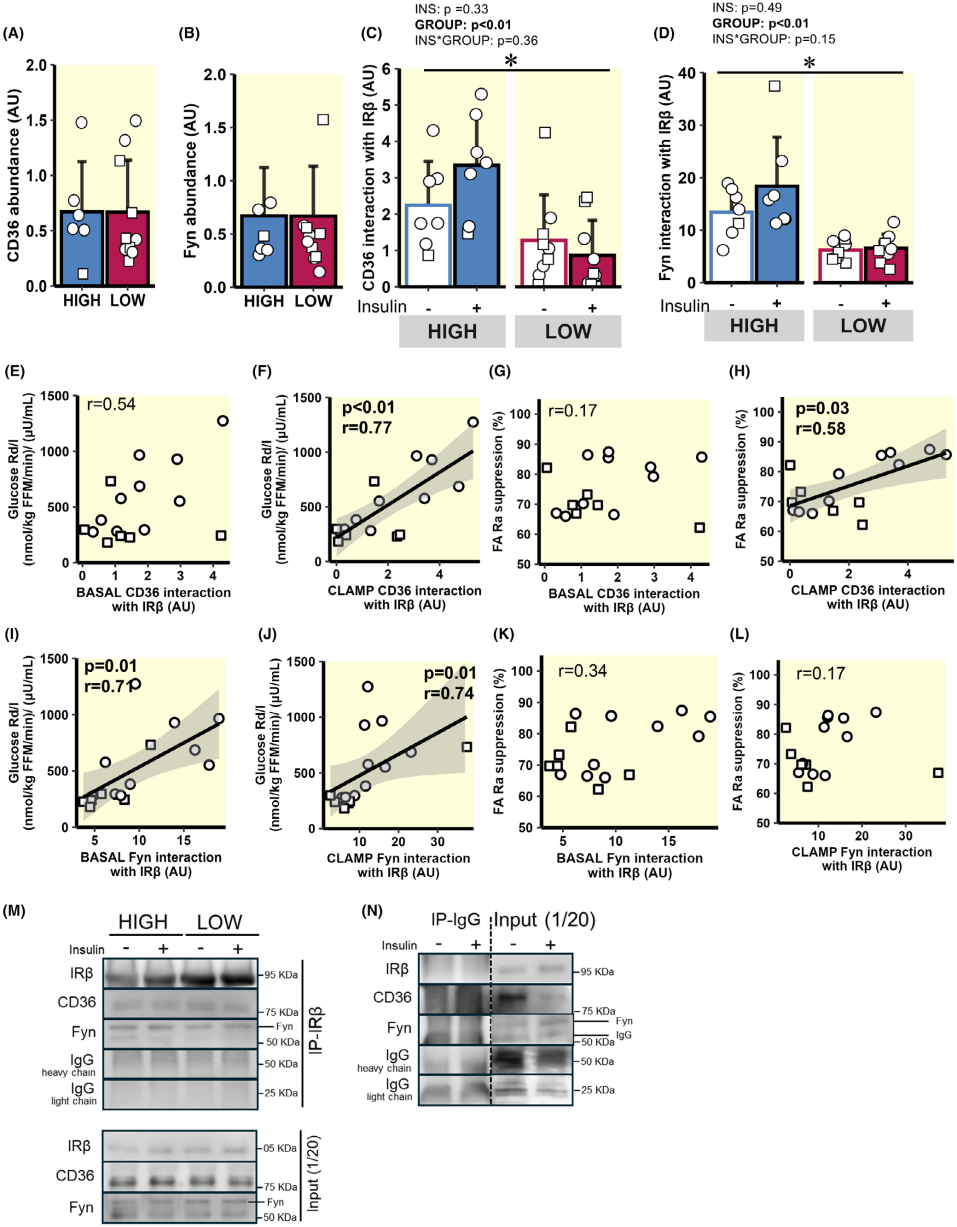
Insulin‐mediated interaction between CD36 and Fyn with IRβ. (A) Whole muscle CD36 abundance. (B) Whole muscle Fyn abundance. (C) CD36 interaction with IRβ at baseline and during the clamp. (D) Fyn interaction with IRβ at baseline and during the clamp. (E and F) Correlation between glucose Rd/I and CD36 interaction with IRβ at baseline (E) and during the clamp (F). (G and H) Correlation between FA Ra suppression and CD36 interaction with IRβ at baseline (G) and during the clamp (H). (I and J) Correlation between glucose Rd/I and Fyn interaction with IRβ at baseline (I) and during the clamp (J). (K and L) Correlation between FA Ra suppression and Fyn interaction with IRβ at baseline (K) and during the clamp (L). (M) Representative immunoblots from the IRβ immunoprecipitation assay, including input controls. (N) IgG immunoprecipitation negative control with dashed lines to denote breaks in lanes removed for clarity. ○ = Female, □ = Male; HIGH versus LOW. *n* = 7 in HIGH and *n* = 9 in LOW. *p < 0.05—main effect for group (LOW versus HIGH). Data are expressed as mean ± SD.

*Skeletal muscle IRβ interaction with CD36 and Fyn*. Total protein abundances for both CD36 and the Src‐family kinase, Fyn, were similar between HIGH and LOW (Figure 4A,B), but the interaction of these proteins with the IRβ was significantly greater in HIGH versus LOW under basal and insulin‐mediated conditions (Figure 4C,D; group main effects; *p* < 0.01). Correlational analyses across all participants revealed that both glucose Rd/I and FA Ra suppression during the clamp were significantly correlated with the interaction of CD36 with IRβ during the clamp (p < 0.01 and *p* = 0.03 respectively; Figure 4F,H), although correlations were not observed under basal conditions (Figure 4E,G). Glucose Rd/I during the clamp was significantly correlated with the interaction of Fyn with IRβ at basal conditions (*p* = 0.01; Figure 4I) and during the clamp (p = 0.01; Figure 4J). In contrast, insulin‐mediated FA Ra suppression was not significantly correlated with the interaction of Fyn with IRβ (Figure 4K,L).

**FIGURE 5:**
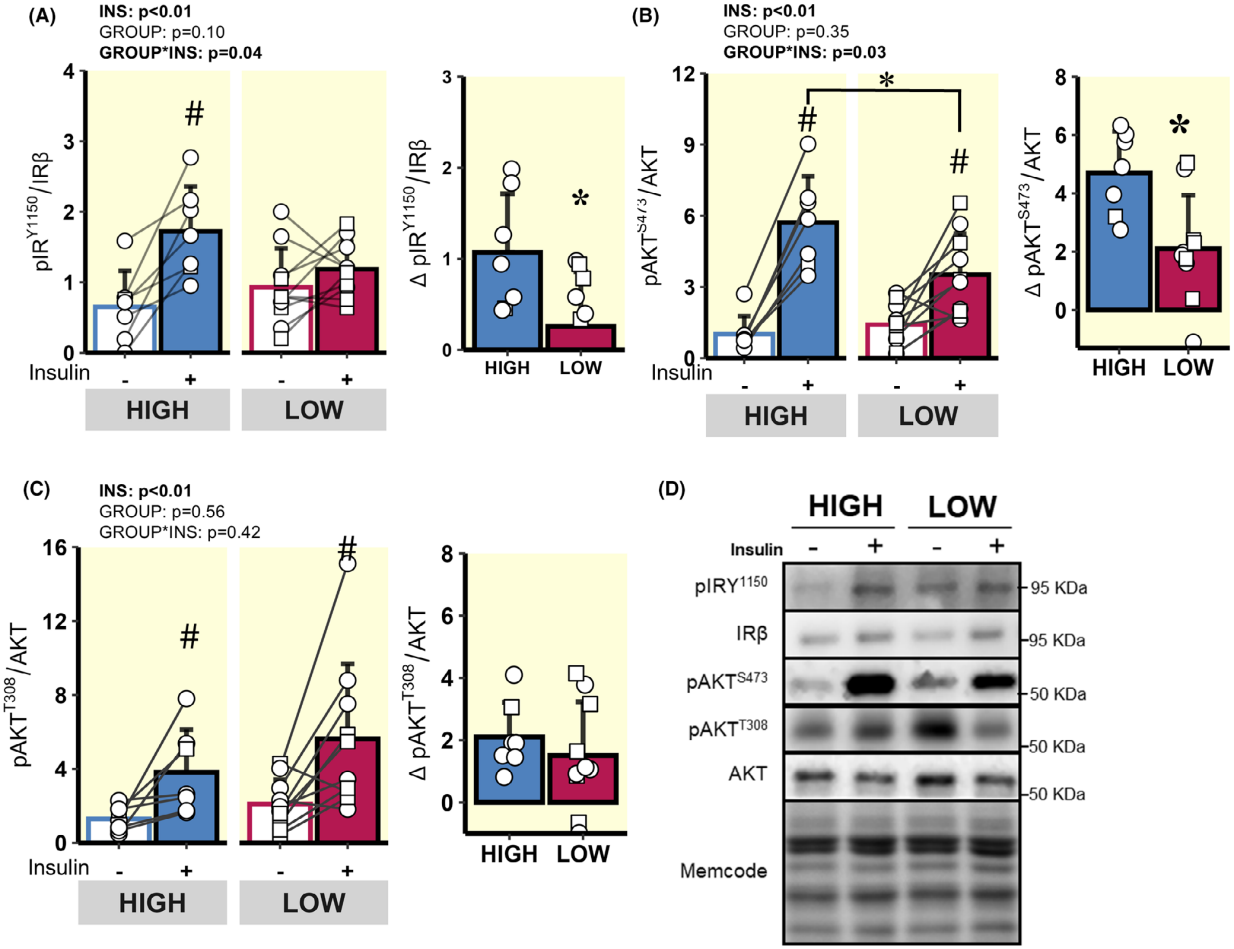
Comparison of acute insulin‐mediated signalling events in skeletal muscle proximal to Akt. (A) pIRβ^Y1150^ before and during the hyperinsulinaemic clamp and Delta pIRβ^Y1150^ in response to insulin (difference between clamp and baseline abundance). (B) pAKT^S473^ before and during the hyperinsulinaemic clamp and Delta pAKT^S473^ in response to insulin. (C) pAKT^T308^ before and during the hyperinsulinaemic clamp and Delta pAKT^T308^ in response to insulin. (D) Representative immunoblots from the whole muscle lysates. ○ = Female, □ = Male; *n* = 7 in HIGH and *n* = 10 in LOW. ^#^ *p* < 0.05 main effect for insulin (basal vs. clamp). *p < 0.05 LOW versus HIGH. Data are expressed as mean ± SD.

*Canonical insulin signalling*. Insulin‐mediated phosphorylation of IRβ at Tyr^1150^ was greater in HIGH versus LOW (Figure 5A), which may align with greater Fyn tyrosine kinase interaction at IRβ. Accordingly, the insulin‐mediated increase in Akt phosphorylation at Ser^473^ was also greater in HIGH versus LOW (p = 0.03; Figure 5B). Insulin‐stimulated Akt phosphorylation at Thr^308^ was not different between HIGH versus LOW groups (Figure 5C).

**FIGURE 6:**
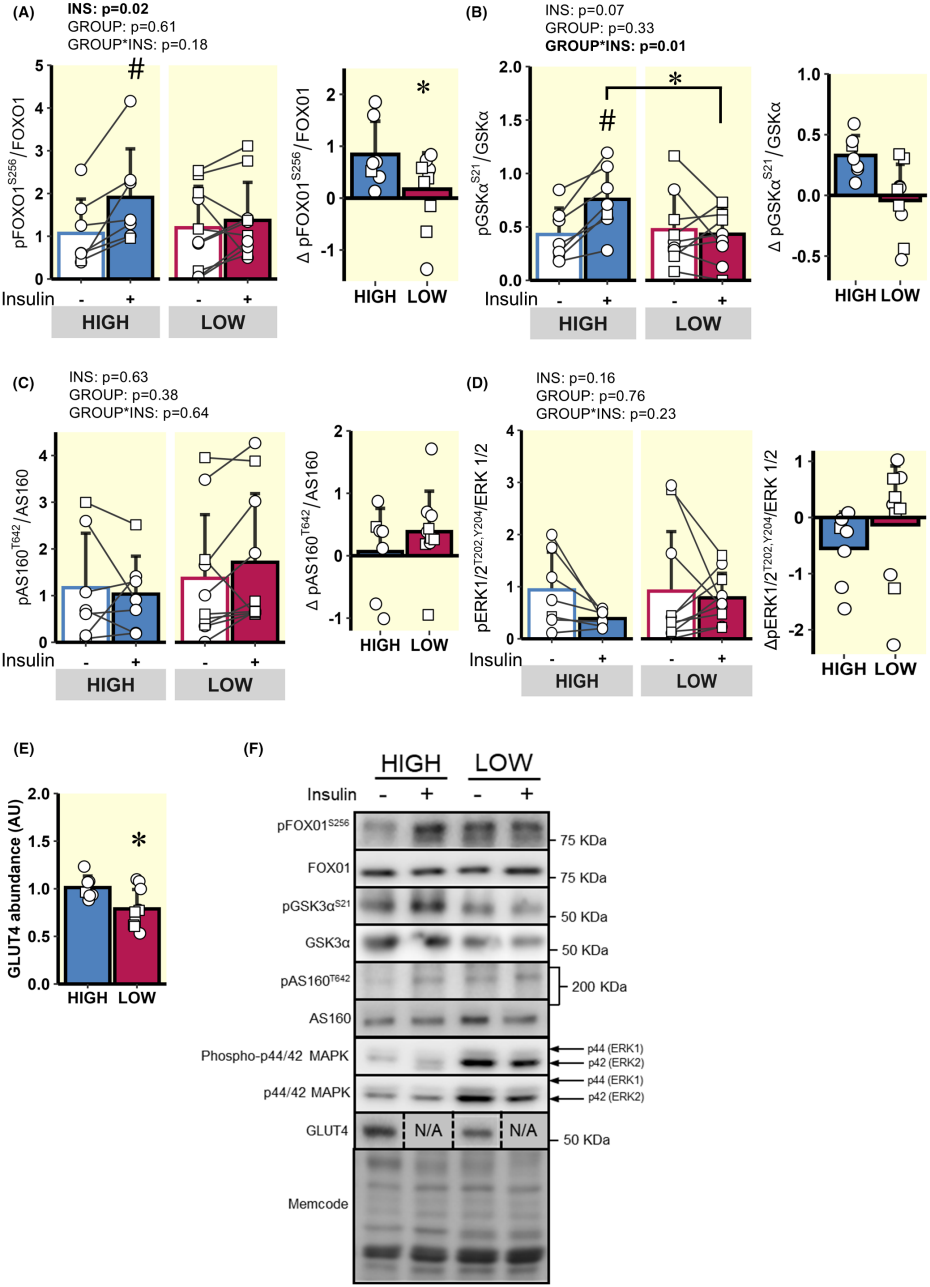
Comparison of acute insulin‐mediated signalling events in skeletal muscle distal to Akt. (A) pFOXO1^S256^ before and during the hyperinsulinaemic clamp and Delta pFOXO1^S256^ in response to insulin. (B) pGSKα^S21^ before and during the hyperinsulinaemic clamp and Delta pGSKα^S21^ in response to insulin. (C) pAS160^T642^ before and during the hyperinsulinaemic clamp and Delta pAS160^T642^ in response to insulin. (D) pERK1/2^T202/Y204^ before and during the hyperinsulinemic clamp and Delta pERK1/2^T202/Y204^ in response to insulin. (E) Skeletal muscle GLUT4 abundance. (F) Representative immunoblots from the whole muscle. ○ = Female, □ = Male; *n* = 7 in HIGH and *n* = 10 in LOW. ^#^ *p* < 0.05 main effect for insulin (basal vs. clamp). *p < 0.05 LOW versus HIGH. Data are expressed as mean ± SD.

Insulin significantly increased FOXO1 phosphorylation at Ser^256^ (*p* = 0.02; Figure 6A) in the HIGH group, and tended to increase GSKα phosphorylation at Ser^21^ (*p* = 0.07; Figure 6B), both of which are downstream targets of Akt. In line with the lower insulin‐mediated increase in Akt phosphorylation at Ser^473^ in LOW versus HIGH, the insulin‐mediated responses for phosphorylation of FOXO1 at Ser^256^ and GSKα at Ser^21^ were also blunted in LOW compared with HIGH (*p* < 0.05; Figure 6A,B). Insulin did not increase AS160 phosphorylation at Thr^642^ or P44/42 MAPK (ERK 1/2) phosphorylation at Thr^202^ and Tyr^204^ in either LOW or HIGH (Figure 6C,D). Interestingly, we found total GLUT4 abundance to be greater in HIGH compared with LOW (*p* = 0.01, Figure 6E).

## 3 DISCUSSION

Although skeletal muscle insulin resistance is very common in adults with obesity,^41^ not all adults with obesity are insulin resistant,^4^, ^5^ and factors underlying this difference among a relatively homogeneous cohort of adults with obesity are not well understood. Here, we confirm that a reduced ability for insulin to suppress FA mobilization from adipose tissue was a strong predictor of impaired insulin‐mediated glucose uptake.^7^, ^8^, ^9^, ^10^ This relationship is consistent with the well‐described relationship that elevated systemic FA availability can lead to a disruption in skeletal muscle insulin signalling.^9^, ^42^, ^43^, ^44^, ^45^ Studies conducted in vitro have reported elevated saturated FA availability reduced the interaction between CD36 and Fyn with IRβ, which in turn modified insulin‐mediated Tyr phosphorylation of IRβ and downstream insulin signalling events.^34^ The interaction between CD36 and Fyn with IRβ was attenuated in skeletal muscle from LOW versus HIGH participants, suggesting that the impairments in skeletal muscle insulin signalling often reported in many with obesity may begin further upstream in the insulin signalling cascade than commonly considered.^13^, ^46^ Additionally, our current findings also suggest differences in the size and location of LDs within skeletal muscle may contribute to differences in insulin‐mediated glucose uptake in a relatively homogeneous cohort of adults with obesity. Together, these findings support the notion that an impaired ability to suppress FA release from adipose tissue in response to insulin may lead to the development of impaired insulin signalling in skeletal muscle, perhaps in part through modifications of LD distribution within skeletal muscle, and interactions between IRβ and key regulatory proteins.

The increase in plasma insulin concentration in response to a meal markedly reduces systemic FA availability in healthy adults.^47^, ^48^ In contrast, individuals who are more resistant to the effects of insulin in adipose tissue experience persistent elevations in systemic FA availability (even after meals), which is known to be a key factor underlying impaired insulin‐mediated glucose uptake in skeletal muscle.^9^, ^44^, ^45^, ^49^ Because the vast majority of FA released into the systemic circulation are derived from aSAT, rather than gluteal/femoral or visceral adipose tissue,^11^ we contend the blunted response to insulin's effect of reducing FA mobilization is primarily occurring in aSAT. Therefore, our findings that a blunted response to insulin's effects on reducing FA mobilization was a primary predictor of impaired insulin‐mediated peripheral glucose uptake, suggests insulin resistance in aSAT may precede the development of insulin resistance in skeletal muscle. However, the temporal pattern by which insulin resistance develops among different tissues is still not clear. Some previous reports in rodents support the notion that the onset of adipose tissue insulin resistance in response to high fat diets occurs before skeletal muscle insulin resistance.^50^, ^51^ For example, in lipodystrophy (a condition where adipose tissue cannot effectively store triglycerides, resulting in very high systemic FA concentrations), the excessive systemic FA availability precedes insulin resistance in skeletal muscle.^52^ In adults with obesity, abnormalities within aSAT include inflammation and fibrosis upon adipocyte expansion are also suggested to contribute to the development of skeletal muscle insulin resistance.^53^, ^54^

The specific mechanisms within aSAT that may underlie an impaired ability for insulin to blunt FA mobilization are not completely understood. Insulin potently inhibits lipolytic rate, largely through Akt‐mediated phosphorylation of phosphodiesterase 3B, which in turn leads to the inhibition of key lipase enzymes.^55^ However, it has also been proposed that low sensitivity to the anti‐lipolytic response to insulin may not be responsible for the blunted FA Ra suppression in response to insulin.^56^ Because adipose tissue is often exceptionally sensitive to the anti‐lipolytic effects of insulin, it is possible that a modest impairment of insulin signalling in aSAT may not lead to a detectable difference in lipolytic rate. Alternatively, a modest impairment in insulin signalling in adipocytes can impact other processes, such as the rate of FA re‐esterification,^57^, ^58^ which may be an important contributor to the blunted FA Ra suppression reported here. Local inflammation (e.g., TNFα, IL6, and IL1β) within adipose tissue has been attributed to impair insulin signalling in adipose tissue,^59^, ^60^, ^61^ and may contribute to impaired ability to reduce FA mobilization in response to insulin. Morphological features of aSAT such as large adipocytes may also contribute to a blunted suppression in FA release, perhaps due in part to greater pro‐inflammatory immune cell infiltration.^62^ In addition, relatively low capillary density coupled with hypertrophied adipocytes can result in attenuated insulin delivery, as well as induce adipocyte hypoxia, which has been linked to impaired insulin responsiveness.^53^, ^54^ Excessive fibrosis within the ECM of aSAT has also been linked with an impaired response to insulin in adipose tissue.^63^, ^64^ How aSAT fibrotic content or composition may reduce insulin signalling within adipose tissue remains elusive, but potential factors include increased expression/production of pro‐inflammatory cytokines by mechanical stress induced by excess collagen accumulation,^65^ and physical constraints limiting insulin delivery to adipocytes.^63^, ^66^ Overall, excessive fibrosis, large adipocyte size, relatively low capillary density, greater inflammatory profile, and attenuated insulin signalling in aSAT may converge to blunt insulin's ability to suppress FA mobilization, and in turn, a resultant chronic elevation in systemic FA may lead to impaired insulin‐mediated glucose uptake in skeletal muscle.

Insulin resistance in skeletal muscle stemming from high FA availability has been causally linked to DAG and ceramide accumulation,^17^, ^21^ resulting in non‐typical PKC isoform activation,^67^ and decreased IRS‐1 and AKT activation.^13^, ^46^, ^68^ However, there remains ongoing debate regarding lipid‐mediated disruption of canonical insulin signalling in skeletal muscle.^24^, ^25^, ^26^ The membrane glycoprotein CD36 is well known for its role in long‐chain FA transport, but recent evidence demonstrated CD36 can also modify insulin signalling.^34^, ^39^ The physical interaction between CD36 and IRβ was found to enhance IRβ phosphorylation by recruiting the Src‐family kinase protein, Fyn, to IRβ where activation via Tyr phosphorylation occurs.^34^ Our findings that the interaction between IRβ and CD36 and Fyn was greater in HIGH versus LOW align with the notion that a greater interaction among these proteins may help propagate intracellular insulin action. Importantly, elevated saturated FA availability was previously found to attenuate CD36 from recruiting Fyn to IRβ, contributing to the blunted insulin‐mediated glucose uptake commonly found when FA availability is high.^34^ Therefore, the reduced ability of insulin to suppress FA mobilization (leading to elevated FA availability) in our LOW participants may have blunted the ability of CD36 to recruit Fyn to IRβ, reducing the interaction between Fyn and IRβ, leading to an attenuation in IRβ phosphorylation. Interestingly, the elevation in plasma insulin concentration during the clamp in our study did not significantly increase the interaction of either CD36 or Fyn with IRβ, suggesting that interactions of these proteins in the basal‐state may be important in the regulation of subsequent IRβ phosphorylation in response to insulin. It is not clear why interactions between IRβ and both CD36 and Fyn were lower in LOW versus HIGH in the basal‐state, especially because basal plasma FA availability was similar between groups. One possibility is that a lower interaction between these proteins may be a chronic adaptation stemming from a persistently elevated FA exposure that LOW subjects may experience if their suppression in FA mobilization in response to insulin is blunted after every meal (similar to our findings during the insulin clamp). Additionally, because insulin is known to trigger intracellular CD36 to the plasma membrane,^69^ the ~threefold higher basal plasma insulin concentrations in LOW versus HIGH may increase in opportunity for FA to interact with CD36, which in turn may attenuate the ability of CD36 to recruit Fyn to IRβ, which has been found to impair downstream insulin signalling.^34^ Our findings that insulin‐mediated phosphorylation of AKT at Ser^473^, as well as phosphorylation of the Akt substrates, GSK3α at Ser and FOXO1 at Ser^256^ were lower in LOW versus HIGH, align with the notion that interaction between IRβ and Fyn may impact canonical insulin signalling downstream of IRβ. Whether the CD36‐mediated recruitment of Fyn to IRβ involves a direct physical interaction between CD36 and Fyn is not known. However, Fyn binding sites have been identified along the cytoplasmic lipid raft domains of CD36,^70^, ^71^ suggesting this process might involve direct binding between these proteins for CD36 to allow phosphorylation of the IRβ by Fyn. Unfortunately, the relatively large amount of tissue required for the co‐immunoprecipitation assay limited our ability to confirm whether CD36 interaction with Fyn was also attenuated in LOW versus HIGH. Together, these findings are the first to our knowledge in human skeletal muscle to support a previously proposed mechanism by which CD36 regulates IRβ phosphorylation^34^ and downstream signalling,^39^ and that FA availability and uptake into skeletal muscle may modify this effect.

Lipid accumulation in skeletal muscle LDs, and LD location within the myocyte have also been implicated in the impairment of insulin‐mediated glucose uptake in skeletal muscle.^72^ Our observation that LDs in the SS region of the myocyte was larger in LOW versus HIGH, aligns with previous reports demonstrating correlations between SS LD size and insulin resistance.^31^, ^32^, ^33^, ^73^ The mechanistic link explaining how the over‐accumulation of larger LDs in the SS region of the myocyte induces insulin resistance is still unclear, but the proximity of these large LDs to the muscle membrane may disrupt key insulin signalling processes.^31^ For example, the accumulation of lipid intermediates in the SS region, which may be released from SS LDs, have been linked with insulin resistance.^16^, ^74^ Additionally, larger LDs have a lower surface area‐to‐volume ratios than small LDs, which may limit lipolytic control at the membrane compared with smaller LDs. In turn, this may increase the susceptibility for incomplete lipolysis,^75^, ^76^, ^77^ resulting in aberrant lipid accumulation within the SS region of the myocyte, in close proximity to insulin signalling events that occur at/near the plasma membrane. Our findings that the number of LDs in the IMF region tended to be greater in HIGH versus LOW suggests that a partitioning of LDs away from the SS region may be protective against impaired insulin signalling. The greater abundance of LDs in the IMF region may also help protect against the development of insulin resistance by their close proximity to more mitochondria, which may aid in the direct lipid transfer toward oxidative metabolism, and thereby may mitigate cytosolic lipid accumulation.^30^, ^78^ We contend that differences in LD size and distribution observed between LOW versus HIGH may be due in part to differences in the ability of insulin to suppress FA release from adipose tissue, whereby, a chronic elevation in systemic FA availability and uptake in individuals with low FA Ra suppression in response to insulin modifies LD storage and metabolism.

Although we interpret our findings describing the relationship between insulin‐mediated suppression of FA release and glucose uptake to suggest a suppressed response to insulin in adipose tissue may contribute the development of whole‐body insulin resistance, we acknowledge that this interpretation was largely based on observational analyses that do not direct causation. However, these findings are consistent with other studies supporting the observation that insulin resistance development is likely a consequence of excess FA release from aSAT,^7^, ^8^, ^9^, ^10^ likely resulting in greater FA uptake into skeletal muscle. We also acknowledge that our in vivo measurements aimed at identifying key contributors to insulin resistance in our participants were not exhaustive, and additional factors may contribute to insulin resistance (e.g., vascular dysfunction limiting insulin delivery)^79^, ^80^ to both skeletal muscle and adipose tissue. Additionally, the phosphorylation of canonical insulin signalling can also be regulated by other intracellular responses that were not measured in this study (e.g., PKCs, JNK, and mTORC1/S6 kinase) – many of which are known to negatively influence activation of proximal insulin signalling components.^81^ It is also important to acknowledge that our LOW group had a greater proportion of male participants compared with HIGH (LOW: 5 M/5F, HIGH: 6F/1M) and men are commonly found to be more insulin resistant than women.^82^, ^83^ Therefore, we cannot exclude the possibility that sex differences are contributing to differences observed in FA release and whole‐body insulin sensitivity between groups. To address this disparity, our statistical approach included ANCOVA analyses to control for sex.

In summary, our findings suggest that a reduced ability to suppress FA mobilization from aSAT in response to insulin is an important contributor to whole‐body insulin resistance. A blunted insulin‐induced suppression of FA mobilization can lead to a persistent elevation in systemic FA availability, which is a major factor underlying the development of insulin resistance in obesity. Participants in our study with relatively low insulin‐mediated glucose uptake also presented larger‐sized LDs in the SS region of the myocyte, which may contribute to their insulin resistance by interfering with key insulin signalling processes at the muscle membrane. Novel findings from our study also suggest this high systemic FA availability may diminish skeletal muscle insulin signalling, in part by reducing the interaction between CD36 and Fyn with IRβ, thereby lowering downstream insulin signalling. Overall, our findings suggest that attenuated suppression in FA mobilization by insulin may contribute to a tissue‐specific crosstalk, whereby elevated FA release from aSAT modifies skeletal muscle LD size and distribution in the myocyte and disrupts IRβ interaction with key regulatory proteins, leading to skeletal muscle insulin resistance.

## 4 MATERIALS AND METHODS

### 4.1 Participants

Seventeen men (*n* = 6) and women (*n* = 11) with obesity participated in this study (BMI = 30–40 kg/m^2^). All participants were “inactive” by not engaging in any planned physical aerobic or resistance exercise and were weight stable (±2 kg) for the previous 6 months. Participants did not take medications known to affect glucose or lipid metabolism, did not have a history of heart disease, and did not actively smoke. All women participating in the study were pre‐menopausal with regularly occurring menses, and were not pregnant or lactating. Participants provided written informed consent before participation. The study conformed to the standards set by the *Declaration of Helsinki*. The study protocol was approved by the University of Michigan Institutional Review Board and was registered at clinicaltrials.gov (NCT02717832). Participants in the present study also participated in a previous study from our laboratory focused on metabolic dysfunction in adipose tissue.^7^ Findings presented here are independent from our previously published work.

### 4.2 Preliminary assessment: body composition, visceral fat area, and liver fat

Body composition was assessed by dual‐energy X‐ray absorptiometry (Lunar DPX DEXA Scanner, GE, Madison, WI) at the Michigan Clinical Unit (MCRU). Visceral fat area (cm^2^) and hepatic fat percentage were measured by magnetic resonance imaging (MRI: Ingenia 3 T MR System, Phillips, Netherlands) at the University of Michigan Medicine's Department of Radiology.

### 4.3 Experimental protocol

The night before the experimental trial, participants were provided a standardized meal at 1900 h and a snack at 2200 h, containing 30% and 10% of their estimated daily energy expenditure, respectively (Figure 1). The macronutrient composition of both the meal and snack were 55% carbohydrate, 30% fat, 15% protein. After the evening snack the participants remained fasted until completion of the study trial the next day. The next morning, participants arrived to the clinical research unit at 0700 h and quietly rested for 60 min. Intravenous catheters were inserted at ~0800 h into a hand vein for blood sampling, and forearm vein for continuous infusions of insulin, glucose, as well as tracer‐labelled solutions of glucose and fatty acid (palmitate). At ~0900 h, a baseline blood sample was collected for background isotope labelling, followed by a primed continuous infusion of [6,6 ^2^H_2_]glucose (35 μmol/kg priming dose, and 0.41 μmol/kg/min continuous infusion; Cambridge Isotopes, Tewksbury, MA). At ~0915 h., a skeletal muscle biopsy was collected from the vastus lateralis muscle, and visible connective tissue and lipid were quickly removed. A portion of each muscle sample was preserved for histology by manually aligning the muscle fibres in parallel under magnification before embedding in optimal cutting temperature (OCT) compound and then flash freezing in isopentane chilled over liquid nitrogen. The remaining portion of the muscle samples were flash frozen in liquid nitrogen for immunoblot analysis. After the basal muscle biopsy, a continuous [1‐^13^C]palmitate bound to human albumin infusion began at ~1000 h (0.04 μmol/kg/min continuous infusion; Cambridge). By ~1050 h, three “arterialized” samples were collected (heated‐hand technique) for measurements of basal glucose and FA kinetics. At ~1100 h, a hyperinsulinemic‐euglycemic clamp procedure began (40 mU insulin/m^2^/min) for determination of whole‐body insulin sensitivity.^84^ Blood samples were collected every 5 min during the clamp to measure blood glucose concentration (StatStrip, Nova Biomedical, Waltham MA) and the infusion rate of dextrose (20% dextrose in 0.9% NaCl) was adjusted accordingly to accommodate insulin‐induced hypoglycemia and attain baseline glucose concentration (~5.0 mM). Exactly 30 min into the hyperinsulinemic–euglycemic clamp procedure, a second muscle biopsy was obtained to identify insulin‐mediated signalling events occurring after acute insulin exposure. After plasma glucose concentration stabilized for a minimum of 20 min without adjusting glucose infusion rate (~120 min into the clamp), arterialized blood samples were collected to quantify insulin‐mediated glucose and FA kinetics.

### 4.4 Insulin‐mediated glucose uptake

Insulin‐mediated glucose uptake was quantified by rate of glucose disappearance from plasma (glucose Rd) at the end of the hyperinsulinemic–euglycemic clamp. Because glucose was in “steady‐state” during the final 20 min of the clamp, glucose Rd at this time was calculated as the sum of the exogenous glucose infusion rate and endogenous hepatic glucose production (glucose rate of appearance in plasma (glucose Ra)). Glucose Rd was then normalized to both fat‐free mass (FFM) and the steady‐state insulin concentration (endogenous insulin production + exogenous insulin infusion) at the end of the clamp (Rd/I = nmol glucose/kg FFM/min/[μU/mL insulin]).

### 4.5 Participant stratification by glucose rate of disappearance

For paired analyses, participants were stratified into “LOW” and “HIGH” cohorts based on their insulin‐sensitivity for glucose uptake: LOW (*n* = 10): glucose Rd/I <400 nmol/kg FFM/min/(μU/mL) and HIGH (*n* = 7): glucose Rd/I >550 nmol/kg FFM/min/(μU/mL). To confirm our measures and participant stratification, HOMA‐IR for all members of the LOW cohort was >2.5, which is a recognized index of peripheral insulin resistance [Table 1, ^85^].

### 4.6 Hepatic and visceral fat

Liver and visceral fat content were measured from MRI images by chemical‐shift‐encoded MRI proton density fat fraction,^86^ and acquisition as previously described by our laboratory.^7^, ^87^ Visceral fat area was measured from three separate axial sections (5 mm thickness) in the L2‐L3 vertebral region, and hepatic fat percentage was measured from three separate axial slices (5 mm thickness) from the liver. Both liver and visceral fat area were quantified by a trained investigator blinded to participant identification.

### 4.7 Plasma glucose and fatty acid kinetics

The tracer‐to‐tracee ratio (TTR) for calculations pertaining to glucose and palmitate kinetics were determined by gas‐chromatography‐mass spectrometry (GC/MS) using a Mass Selective Detector 5973 system (Agilent Technologies, Santa Clara, CA), as described by our laboratory.^7^, ^88^ Palmitate Ra and glucose Ra were calculated from their respective TTR using the Steele equation for steady state conditions.^89^ FA Ra was quantified by dividing palmitate Ra by the ratio of total palmitate (C16:0) to FAs within a standard. Because samples were collected during steady‐state conditions, rates of disappearance from the circulation were equal to their rates of appearance into the circulation (i.e., Ra = Rd).

### 4.8 Plasma glucose, lipid, and insulin concentration

Plasma glucose, non‐esterified fatty acid, triacylglycerol, high‐density lipoprotein (HDL), and total cholesterol were measured in plasma from commercially available colorimetric assays. Plasma interleukin‐6 (IL‐6), C‐reactive protein (CRP), leptin, total adiponectin, and high molecular weight (HMW) adiponectin were measured via enzyme‐linked immunoassay (ELISA; R&D systems). Insulin was measured in serum by radio‐immunoassay (Siemens). See Table S1 for vendor details.

### 4.9 Skeletal muscle histochemistry: LD processing and fluorescence image acquisition and analysis

Muscle samples from OCT embedded tissues were cut into 5 μm sections at −20°C and mounted onto an ethanol‐cleaned glass slide. Sections were fixed in 4% paraformaldehyde for 60 min, and permeabilized for 5 min in 0.5% triton X‐100. Sections were then incubated with primary antibodies myosin heavy chain type 1 (MHC‐1) at 1:200 for 60 min at 37°C, followed by 30 min secondary antibody incubation with AlexaFluor 647, goat‐anti mouse IgG2b at 1:200. Afterward, sections were stained with Bodipy 493/503 at 1:100 for 20 min, followed by 20 min incubation with anti‐wheat germ agglutinin (WGA) conjugated with AlexaFluor 555 at 1:500 to identify muscle membranes. Sections were mounted with ProLong Gold Antifade Mountant, covered with #1 coverslips (2975–245, Corning, Corning, NY), and stored in the dark until imaging. See Table S1 for detailed histochemistry vendor details.

Images used for the quantification of LD area (% stained), LD number (number of LDs/mm^2^), and median LD size (μm^2^) were captured on a Keyence Bz‐X710 fluorescence microscope (Keyence; Osaka Japan) with a x20 N.A. = 0.45 objective lens using 1440 X 1080 pixels (533 μm x 400 μm) field of view. After image acquisition, LD characteristics (i.e., number and size), and distribution were quantified using MATLAB R2021a (Mathworks, Inc., Natick, MA). In brief, images captured at x20 were converted to greyscale and re‐scaled to accommodate image variability between samples. Individual muscle fibres were identified and labelled using a custom ridge detection algorithm and watershed segmentation to complete non‐continuous cell borders and completely identify cells. Type I fibres were identified based on positive MHC type I‐positive stain, and non‐stained fibres considered type II fibres. Fibre types were partitioned into type I or type II fibres dependent on signal intensity using k‐means clustering (k = 2). Lipid droplets identified in the SS region were contained within ~3 μm of the peripheral border of each fibre, while the IMF region remained toward the center of each fibre (~91% of total myocyte volume). Lipid droplets were detected as the mean area of puncta, and a top‐hat filter was used to accommodate for image variability and background noise produced by light scatter of the fluorescence microscope. Lipid droplets identified and included for analysis within each muscle fibre were contained within a normal Gaussian distribution. In total, 3–5 fields of view were obtained per participant, resulting in the analysis of 125 ± 57 muscle fibres per participant.

### 4.10 Skeletal muscle lysate preparation for immunoblot

Frozen muscle samples were weighed (~25 mg) and transferred into pre‐chilled microcentrifuge tubes containing 1 mL ice‐cold lysis buffer (RIPA: 20 mM Tris–HCl‐pH 7.5, 150 mM NaCl, 1 mM Na_2_EDTA, 1 mM EGTA, 1% NP‐40, 1% sodium deoxycholate, 2.5 mM sodium pyrophosphate, 1 mM β‐glycerophosphate, 1 mM Na_3_VO_4_, 1 μg/mL leupeptin [9086, Cell Signaling Technology, Danvers, MA]), 1% phosphatase inhibitor cocktail #1 & #2 (P2850, P5726, Sigma‐Aldrich, St. Louis, MS), 1% protease inhibitor cocktail (P8340, Sigma‐Aldrich), and two steel ball bearings per sample. Samples were homogenized for 30s at 45 Hz using Qiagen TissueLyser II, then solubilized for 60 min by inverted end‐over‐end rotation at 50 rpm in the cold (4°C). Afterward, samples were centrifuged at 15 000*g* for 15 min in 4°C, discarding the pellet. Protein concentration was determined by bicinchoninic acid (BCA) assay (23225, Thermo‐Fisher, Waltham, MA), and samples were diluted in 4X Laemmli buffer (1610747, Bio‐Rad, Hercules, CA) to 1 mg/mL concentration, then heated for 7 min in 95°C. Proteins were separated by SDS‐Page (8–12% acrylamide), transferred to nitrocellulose membranes, and probed for the primary antibodies described in Table S1. To account for loading control, membranes were normalized to total protein abundance determined by Memcode reversible protein stain (Pierce Thermo‐Fisher). Additionally, each gel contained an internal standard sample from eight obese individuals to account for gel‐to‐gel variability, in which each sample was also normalized to their internal standard.

### 4.11 Immunoprecipitation

Frozen skeletal muscle samples were homogenized in 1% triton‐based lysis buffer (20 mM Tris–HCl (pH 7.5), 150 mM NaCl, 1 mM Na2EDTA, 1 mM EGTA, 1% triton X‐100, 2.5 mM sodium pyrophosphate, 1 mM beta‐glycerophosphate, 1 mM Na3VO4, 1 μg/mL leupeptin; 9803, Cell Signaling Technology) using a chilled dounce homogenizer. Homogenates were then solubilized by end‐over‐end rotation for 60 min and centrifuged at 100 000*g* for 60 min to limit sarcomere proteins. The supernatant was then collected, protein concentration quantified (BCA), and 300 μg protein from the lysate was adjusted to 500 μL volume with lysis buffer. The adjusted volume was rotated end‐over‐end overnight at 4°C with 5 μg IRβ antibody. The next day, 25 μL protein‐A magnetic beads (88845, Pierce Thermo‐Fisher) were washed in wash buffer (25 mM Tris, 0.15 M NaCl, 0.05% Tween‐20, pH 7.5), then added to the lysate‐antibody mixture, and rotated gently end‐over‐end for 120 min at room temperature. The immunoprecipitation matrix was separated using a magnetic rack (DynaMag2, 12321D, Thermo‐Fisher) and washed three times with lysis buffer, twice with wash buffer, and once with PBS. Antigens were eluted from the bead complex with 80 μL 2X Laemmli SDS buffer, by a combination of rotation and vortexing samples for 10 min and discarding the beads following protein elution. After immunoprecipitation, protein abundance from the eluates was analysed by immunoblot for basal and insulin‐mediated CD36, IRβ, and Fyn. CD36 and Fyn interaction with IRβ was normalized to a 15 μL input sample from the same participant loaded onto the same gel as the eluate.

### 4.12 Statistical analysis

All measurements that were not normally distributed were log‐transformed. A linear mixed model analysis of covariance (ANCOVA) was conducted to assess the primary effects of the different groups (HIGH vs. LOW), the effects of insulin‐stimulation (basal vs. insulin‐mediated conditions), and any interactions between group and insulin. This analysis was controlled for sex as a covariate for measurements and included targeted protein analysis, immunoprecipitation, FA Ra, and glucose Ra. For LD histochemistry analysis, measurements of lipid content, LD number, and LD area were segregated into regional distribution (IMF and SS region). A linear mixed model ANCOVA was then used on the segregated outcomes to identify main effects of group (HIGH vs. LOW), main effects for fibre type (type I vs. type II), and group x fibre‐type interaction in the IMF and SS region independently, with sex as a covariate. When significant interactions were observed, Tukey's post‐hoc analysis was used to identify significant interactions between groups. Unpaired Student's *t*‐tests were used to test for significant differences between groups (HIGH vs. LOW) for all other clinical variables. Multiple linear regression was used to identify relationships between clinical outcomes and glucose Rd/I, adjusted for sex as a covariate. Statistical analysis was completed using R version 4.1.0. Data are displayed as mean ± SD, and significance set to *p* < 0.05.

## 5 CONCLUSION

In conclusion, our findings suggest that reduced ability for insulin to suppress fatty acid mobilization with accompanying changes in lipid droplet size and distribution within the muscle cell, along with attenuated interaction between the insulin receptor and CD36 and Fyn in skeletal muscle, may be key contributors to the impaired insulin‐mediated glucose uptake commonly found in adults with obesity.

## AUTHOR CONTRIBUTIONS

**Michael W. Schleh:** Data curation; investigation; formal analysis; writing – original draft; writing – review and editing; visualization. **Benjamin J. Ryan:** Data curation; methodology; formal analysis; supervision; project administration; writing – review and editing. **Cheehoon Ahn:** Data curation; investigation; formal analysis; writing – review and editing. **Alison C. Ludzki:** Data curation; methodology; project administration. **Douglas W. Van Pelt:** Methodology; data curation; investigation; project administration. **Lisa M. Pitchford:** Data curation; investigation; project administration; methodology. **Olivia K. Chugh:** Investigation. **Austin T. Luker:** Investigation. **Kathryn E. Luker:** Investigation. **Dmitri Samovski:** Conceptualization. **Nada A. Abumrad:** Conceptualization. **Charles F. Burant:** Supervision; project administration. **Jeffrey F. Horowitz:** Conceptualization; methodology; data curation; investigation; supervision; funding acquisition; project administration; writing – review and editing.

## FUNDING INFORMATION

National Institutes of Health (R01DK077966, P30DK089503, U24DK097153, T32DK007245, F32DK117522), American Diabetes Association (1‐16‐ICTS‐048), and the Canadian Institutes of Health Research (146190).

## CONFLICT OF INTEREST STATEMENT

None of the authors have a conflict of interest to disclose.

## Supporting information


Table S1.

## Data Availability

The authors confirm that the data supporting the findings of this study are available within the article and upon request with the corresponding author.

## References

[1] Hales CM , Carroll MD , Fryar CD , Ogden CL . Prevalence of obesity and severe obesity among adults: United States, 2017–2018. NCHS Data Brief. 2020;360:1‐8. 32487284

[2] Farin HM , Abbasi F , Reaven GM . Body mass index and waist circumference both contribute to differences in insulin‐mediated glucose disposal in nondiabetic adults. Am J Clin Nutr. 2006;83(1):47‐51. 16400048 10.1093/ajcn/83.1.47

[3] Reaven GM . Banting lecture 1988. Role of insulin resistance in human disease. Diabetes. 1988;37(12):1595‐1607. 3056758 10.2337/diab.37.12.1595

[4] Smith GI , Mittendorfer B , Klein S . Metabolically healthy obesity: facts and fantasies. J Clin Invest. 2019;129(10):3978‐3989. 31524630 10.1172/JCI129186 PMC6763224

[5] Wildman RP , Muntner P , Reynolds K , et al. The obese without cardiometabolic risk factor clustering and the normal weight with cardiometabolic risk factor clustering: prevalence and correlates of 2 phenotypes among the US population (NHANES 1999‐2004). Arch Intern Med. 2008;168(15):1617‐1624. 18695075 10.1001/archinte.168.15.1617

[6] Van Pelt DW , Newsom SA , Schenk S , Horowitz JF . Relatively low endogenous fatty acid mobilization and uptake helps preserve insulin sensitivity in obese women. Int J Obes. 2015;39(1):149‐155. 10.1038/ijo.2014.70 PMC4216778 24785103

[7] Schleh MW , Ryan BJ , Ahn C , et al. Metabolic dysfunction in obesity is related to impaired suppression of fatty acid release from adipose tissue by insulin. Obesity (Silver Spring). 2023;31(5):1347‐1361. 36988872 10.1002/oby.23734 PMC10192005

[8] Magkos F , Fabbrini E , Conte C , Patterson BW , Klein S . Relationship between adipose tissue lipolytic activity and skeletal muscle insulin resistance in nondiabetic women. J Clin Endocrinol Metab. 2012;97(7):E1219‐E1223. 22492868 10.1210/jc.2012-1035 PMC3387393

[9] Groop LC , Bonadonna RC , DelPrato S , et al. Glucose and free fatty acid metabolism in non‐insulin‐dependent diabetes mellitus. Evidence for multiple sites of insulin resistance. J Clin Invest. 1989;84(1):205‐213. 2661589 10.1172/JCI114142 PMC303971

[10] Koh H‐CE , van Vliet S , Pietka TA , et al. Subcutaneous adipose tissue metabolic function and insulin sensitivity in people with obesity. Diabetes. 2021;70(10):2225‐2236. 34266892 10.2337/db21-0160 PMC8576507

[11] Nielsen S , Guo Z , Johnson CM , Hensrud DD , Jensen MD . Splanchnic lipolysis in human obesity. J Clin Invest. 2004;113(11):1582‐1588. 15173884 10.1172/JCI21047 PMC419492

[12] Krssak M , Falk Petersen K , Dresner A , et al. Intramyocellular lipid concentrations are correlated with insulin sensitivity in humans: a 1H NMR spectroscopy study. Diabetologia. 1999;42(1):113‐116. 10027589 10.1007/s001250051123

[13] Dresner A , Laurent D , Marcucci M , et al. Effects of free fatty acids on glucose transport and IRS‐1‐associated phosphatidylinositol 3‐kinase activity. J Clin Invest. 1999;103(2):253‐259. 9916137 10.1172/JCI5001 PMC407880

[14] Weiss R , Dufour S , Taksali SE , et al. Prediabetes in obese youth: a syndrome of impaired glucose tolerance, severe insulin resistance, and altered myocellular and abdominal fat partitioning. Lancet. 2003;362(9388):951‐957. 14511928 10.1016/S0140-6736(03)14364-4 PMC2995523

[15] DeVito LM , Dennis EA , Kahn BB , et al. Bioactive lipids and metabolic syndrome—a symposium report. Ann N Y Acad Sci. 2022;1511(1):87‐106. 35218041 10.1111/nyas.14752 PMC9219555

[16] Song JD , Alves TC , Befroy DE , et al. Dissociation of muscle insulin resistance from alterations in mitochondrial substrate preference. Cell Metab. 2020;32(5):726‐735.e5. 33035493 10.1016/j.cmet.2020.09.008 PMC8218871

[17] Adams JM , Pratipanawatr T , Berria R , et al. Ceramide content is increased in skeletal muscle from obese insulin‐resistant humans. Diabetes. 2004;53(1):25‐31. 14693694 10.2337/diabetes.53.1.25

[18] Petersen MC , Smith GI , Palacios HH , et al. Cardiometabolic characteristics of people with metabolically healthy and unhealthy obesity. Cell Metab. 2024;36(4):745‐761.e5. 38569471 10.1016/j.cmet.2024.03.002 PMC11025492

[19] Koves TR , Ussher JR , Noland RC , et al. Mitochondrial overload and incomplete fatty acid oxidation contribute to skeletal muscle insulin resistance. Cell Metab. 2008;7(1):45‐56. 18177724 10.1016/j.cmet.2007.10.013

[20] Houmard JA , Tanner CJ , Yu C , et al. Effect of weight loss on insulin sensitivity and intramuscular long‐chain fatty acyl‐CoAs in morbidly obese subjects. Diabetes. 2002;51(10):2959‐2963. 12351433 10.2337/diabetes.51.10.2959

[21] Itani SI , Ruderman NB , Schmieder F , Boden G . Lipid‐induced insulin resistance in human muscle is associated with changes in diacylglycerol, protein kinase C, and IκB‐α. Diabetes. 2002;51(7):2005‐2011. 12086926 10.2337/diabetes.51.7.2005

[22] Batista TM , Haider N , Kahn CR . Defining the underlying defect in insulin action in type 2 diabetes. Diabetologia. 2021;64(5):994‐1006. 33730188 10.1007/s00125-021-05415-5 PMC8916220

[23] Moeschel K , Beck A , Weigert C , et al. Protein kinase C‐zeta‐induced phosphorylation of Ser318 in insulin receptor substrate‐1 (IRS‐1) attenuates the interaction with the insulin receptor and the tyrosine phosphorylation of IRS‐1. J Biol Chem. 2004;279(24):25157‐25163. 15069075 10.1074/jbc.M402477200

[24] Hoy AJ , Brandon AE , Turner N , et al. Lipid and insulin infusion‐induced skeletal muscle insulin resistance is likely due to metabolic feedback and not changes in IRS‐1, Akt, or AS160 phosphorylation. Am J Physiol Endocrinol Metab. 2009;297(1):E67‐E75. 19366875 10.1152/ajpendo.90945.2008 PMC2711668

[25] Skovbro M , Baranowski M , Skov‐Jensen C , et al. Human skeletal muscle ceramide content is not a major factor in muscle insulin sensitivity. Diabetologia. 2008;51(7):1253‐1260. 18458871 10.1007/s00125-008-1014-z

[26] Timmers S , Nabben M , Bosma M , et al. Augmenting muscle diacylglycerol and triacylglycerol content by blocking fatty acid oxidation does not impede insulin sensitivity. Proc Natl Acad Sci USA. 2012;109(29):11711‐11716. 22753483 10.1073/pnas.1206868109 PMC3406830

[27] Walther TC , Farese RV Jr . Lipid droplets and cellular lipid metabolism. Annu Rev Biochem. 2012;81:687‐714. 22524315 10.1146/annurev-biochem-061009-102430 PMC3767414

[28] Olzmann JA , Carvalho P . Dynamics and functions of lipid droplets. Nat Rev Mol Cell Biol. 2019;20(3):137‐155. 30523332 10.1038/s41580-018-0085-z PMC6746329

[29] Ferreira R , Vitorino R , Alves RM , et al. Subsarcolemmal and intermyofibrillar mitochondria proteome differences disclose functional specializations in skeletal muscle. Proteomics. 2010;10(17):3142‐3154. 20665633 10.1002/pmic.201000173

[30] Shaw CS , Jones DA , Wagenmakers AJM . Network distribution of mitochondria and lipid droplets in human muscle fibres. Histochem Cell Biol. 2008;129(1):65‐72. 17938948 10.1007/s00418-007-0349-8

[31] Daemen S , Gemmink A , Brouwers B , et al. Distinct lipid droplet characteristics and distribution unmask the apparent contradiction of the athlete's paradox. Mol Metab. 2018;17:71‐81. 30174227 10.1016/j.molmet.2018.08.004 PMC6197504

[32] Nielsen J , Christensen AE , Nellemann B , Christensen B . Lipid droplet size and location in human skeletal muscle fibers are associated with insulin sensitivity. Am J Physiol Endocrinol Metab. 2017;313(6):E721‐E730. 28743757 10.1152/ajpendo.00062.2017

[33] Nielsen J , Mogensen M , Vind BF , et al. Increased subsarcolemmal lipids in type 2 diabetes: effect of training on localization of lipids, mitochondria, and glycogen in sedentary human skeletal muscle. Am J Physiol Endocrinol Metab. 2010;298(3):E706‐E713. 20028967 10.1152/ajpendo.00692.2009

[34] Samovski D , Dhule P , Pietka T , et al. Regulation of insulin receptor pathway and glucose metabolism by CD36 signaling. Diabetes. 2018;67(7):1272‐1284. 29748289 10.2337/db17-1226 PMC6014550

[35] Pepino MY , Kuda O , Samovski D , Abumrad NA . Structure‐function of CD36 and importance of fatty acid signal transduction in fat metabolism. Annu Rev Nutr. 2014;34:281‐303. 24850384 10.1146/annurev-nutr-071812-161220 PMC4329921

[36] Su X , Abumrad NA . Cellular fatty acid uptake: a pathway under construction. Trends Endocrinol Metab. 2009;20(2):72‐77. 19185504 10.1016/j.tem.2008.11.001 PMC2845711

[37] Samovski D , Jacome‐Sosa M , Abumrad NA . Fatty acid transport and signaling: mechanisms and physiological implications. Annu Rev Physiol. 2023;85(1):317‐337. 36347219 10.1146/annurev-physiol-032122-030352 PMC13221695

[38] Glatz JC , Luiken JFP . Dynamic role of the transmembrane glycoprotein CD36 (SR‐B2) in cellular fatty acid uptake and utilization. J Lipid Res. 2018;59(7):1084‐1093. 29627764 10.1194/jlr.R082933 PMC6027920

[39] Sun S , Tan P , Huang X , et al. Ubiquitinated CD36 sustains insulin‐stimulated Akt activation by stabilizing insulin receptor substrate 1 in myotubes. J Biol Chem. 2018;293(7):2383‐2394. 29269414 10.1074/jbc.M117.811471 PMC5818186

[40] Samovski D , Sun J , Pietka T , et al. Regulation of AMPK activation by CD36 links fatty acid uptake to β‐oxidation. Diabetes. 2015;64(2):353‐359. 25157091 10.2337/db14-0582 PMC4303974

[41] DeFronzo RA , Ferrannini E . Insulin resistance: a multifaceted syndrome responsible for NIDDM, obesity, hypertension, dyslipidemia, and atherosclerotic cardiovascular disease. Diabetes Care. 1991;14(3):173‐194. 2044434 10.2337/diacare.14.3.173

[42] Roden M , Price TB , Perseghin G , et al. Mechanism of free fatty acid‐induced insulin resistance in humans. J Clin Invest. 1996;97(12):2859‐2865. 8675698 10.1172/JCI118742 PMC507380

[43] Roden M , Stingl H , Chandramouli V , et al. Effects of free fatty acid elevation on postabsorptive endogenous glucose production and gluconeogenesis in humans. Diabetes. 2000;49(5):701‐707. 10905476 10.2337/diabetes.49.5.701

[44] Boden G . Role of fatty acids in the pathogenesis of insulin resistance and NIDDM. Diabetes. 1997;46(1):3‐10. 8971073

[45] Thiébaud D , DeFronzo RA , Jacot E , et al. Effect of long chain triglyceride infusion on glucose metabolism in man. Metabolism. 1982;31(11):1128‐1136. 6752642 10.1016/0026-0495(82)90163-9

[46] Li Y , Soos TJ , Li X , et al. Protein kinase C θ inhibits insulin signaling by phosphorylating IRS1 at Ser1101. J Biol Chem. 2004;279(44):45304‐45307. 15364919 10.1074/jbc.C400186200

[47] Coppack SW , Fisher RM , Gibbons GF , et al. Postprandial substrate deposition in human forearm and adipose tissues in vivo. Clin Sci (Lond). 1990;79(4):339‐348. 2171854 10.1042/cs0790339

[48] Frayn KN . Adipose tissue as a buffer for daily lipid flux. Diabetologia. 2002;45(9):1201‐1210. 12242452 10.1007/s00125-002-0873-y

[49] Roden M , Krssak M , Stingl H , et al. Rapid impairment of skeletal muscle glucose transport/phosphorylation by free fatty acids in humans. Diabetes. 1999;48(2):358‐364. 10334314 10.2337/diabetes.48.2.358

[50] Turner N , Kowalski GM , Leslie SJ , et al. Distinct patterns of tissue‐specific lipid accumulation during the induction of insulin resistance in mice by high‐fat feeding. Diabetologia. 2013;56(7):1638‐1648. 23620060 10.1007/s00125-013-2913-1

[51] Burchfield JG , Kebede MA , Meoli CC , et al. High dietary fat and sucrose result in an extensive and time‐dependent deterioration in health of multiple physiological systems in mice. J Biol Chem. 2018;293(15):5731‐5745. 29440390 10.1074/jbc.RA117.000808 PMC5900752

[52] Mann JP , Savage DB . What lipodystrophies teach us about the metabolic syndrome. J Clin Invest. 2019;129(10):4009‐4021. 31380809 10.1172/JCI129190 PMC6763226

[53] Cifarelli V , Beeman SC , Smith GI , et al. Decreased adipose tissue oxygenation associates with insulin resistance in individuals with obesity. J Clin Invest. 2020;130(12):6688‐6699. 33164985 10.1172/JCI141828 PMC7685757

[54] Crewe C , An YA , Scherer PE . The ominous triad of adipose tissue dysfunction: inflammation, fibrosis, and impaired angiogenesis. J Clin Invest. 2017;127(1):74‐82. 28045400 10.1172/JCI88883 PMC5199684

[55] DiPilato LM , Ahmad F , Harms M , Seale P , Manganiello V , Birnbaum MJ . The role of PDE3B phosphorylation in the inhibition of lipolysis by insulin. Mol Cell Biol. 2015;35(16):2752‐2760. 26031333 10.1128/MCB.00422-15 PMC4508315

[56] Yeckel CW , Dziura J , DiPietro L . Abdominal obesity in older women: potential role for disrupted fatty acid reesterification in insulin resistance. J Clin Endocrinol Metab. 2008;93(4):1285‐1291. 18198225 10.1210/jc.2007-1875 PMC2291490

[57] Meegalla RL , Billheimer JT , Cheng D . Concerted elevation of acyl‐coenzyme a:diacylglycerol acyltransferase (DGAT) activity through independent stimulation of mRNA expression of DGAT1 and DGAT2 by carbohydrate and insulin. Biochem Biophys Res Commun. 2002;298(3):317‐323. 12413942 10.1016/s0006-291x(02)02466-x

[58] Ali AH , Mundi M , Koutsari C , Bernlohr DA , Jensen MD . Adipose tissue free fatty acid storage in vivo: effects of insulin versus niacin as a control for suppression of lipolysis. Diabetes. 2015;64(8):2828‐2835. 25883112 10.2337/db14-1409 PMC4512218

[59] Lagathu C , Yvan‐Charvet L , Bastard JP , et al. Long‐term treatment with interleukin‐1β induces insulin resistance in murine and human adipocytes. Diabetologia. 2006;49(9):2162‐2173. 16865359 10.1007/s00125-006-0335-z

[60] Hotamisligil GS , Peraldi P , Budavari A , Ellis R , White MF , Spiegelman BM . IRS‐1‐mediated inhibition of insulin receptor tyrosine kinase activity in TNF‐alpha‐ and obesity‐induced insulin resistance. Science. 1996;271(5249):665‐668. 8571133 10.1126/science.271.5249.665

[61] Lagathu C , Bastard J‐P , Auclair M , Maachi M , Capeau J , Caron M . Chronic interleukin‐6 (IL‐6) treatment increased IL‐6 secretion and induced insulin resistance in adipocyte: prevention by rosiglitazone. Biochem Biophys Res Commun. 2003;311(2):372‐379. 14592424 10.1016/j.bbrc.2003.10.013

[62] Guilherme A , Virbasius JV , Puri V , Czech MP . Adipocyte dysfunctions linking obesity to insulin resistance and type 2 diabetes. Nat Rev Mol Cell Biol. 2008;9(5):367‐377. 18401346 10.1038/nrm2391 PMC2886982

[63] Marcelin G , Ferreira A , Liu Y , et al. A PDGFRα‐mediated switch toward CD9(high) adipocyte progenitors controls obesity‐induced adipose tissue fibrosis. Cell Metab. 2017;25(3):673‐685. 28215843 10.1016/j.cmet.2017.01.010

[64] Khan T , Muise ES , Iyengar P , et al. Metabolic dysregulation and adipose tissue fibrosis: role of collagen VI. Mol Cell Biol. 2009;29(6):1575‐1591. 19114551 10.1128/MCB.01300-08 PMC2648231

[65] Pellegrinelli V , Heuvingh J , du Roure O , et al. Human adipocyte function is impacted by mechanical cues. J Pathol. 2014;233(2):183‐195. 24623048 10.1002/path.4347

[66] Marcelin G , Silveira ALM , Martins LB , Ferreira AVM , Clément K . Deciphering the cellular interplays underlying obesity‐induced adipose tissue fibrosis. J Clin Invest. 2019;129(10):4032‐4040. 31498150 10.1172/JCI129192 PMC6763252

[67] Itani SI , Zhou Q , Pories WJ , MacDonald KG , Dohm GL . Involvement of protein kinase C in human skeletal muscle insulin resistance and obesity. Diabetes. 2000;49(8):1353‐1358. 10923637 10.2337/diabetes.49.8.1353

[68] Dobrowsky RT , Kamibayashi C , Mumby MC , Hannun YA . Ceramide activates heterotrimeric protein phosphatase 2A. J Biol Chem. 1993;268(21):15523‐15530. 8393446

[69] Luiken JJFP , Dyck DJ , Han X‐X , et al. Insulin induces the translocation of the fatty acid transporter FAT/CD36 to the plasma membrane. Am J Physiol Endocrinol Metab. 2002;282(2):E491‐E495. 11788383 10.1152/ajpendo.00419.2001

[70] Huang MM , Bolen JB , Barnwell JW , Shattil SJ , Brugge JS . Membrane glycoprotein IV (CD36) is physically associated with the Fyn, Lyn, and yes protein‐tyrosine kinases in human platelets. Proc Natl Acad Sci USA. 1991;88(17):7844‐7848. 1715582 10.1073/pnas.88.17.7844 PMC52400

[71] Bull HA , Brickell PM , Dowd PM . Src‐related protein tyrosine kinases are physically associated with the surface antigen CD36 in human dermal microvascular endothelial cells. FEBS Lett. 1994;351(1):41‐44. 7521304 10.1016/0014-5793(94)00814-0

[72] Gemmink A , Goodpaster BH , Schrauwen P , Hesselink MKC . Intramyocellular lipid droplets and insulin sensitivity, the human perspective. Biochim Biophys Acta Mol Cell Biol Lipids. 2017;1862(10, Part B):1242‐1249. 28739280 10.1016/j.bbalip.2017.07.010

[73] Chee C , Shannon CE , Burns A , et al. Relative contribution of intramyocellular lipid to whole‐body fat oxidation is reduced with age but subsarcolemmal lipid accumulation and insulin resistance are only associated with overweight individuals. Diabetes. 2016;65(4):840‐850. 26740597 10.2337/db15-1383 PMC4894456

[74] Perreault L , Newsom SA , Strauss A , et al. Intracellular localization of diacylglycerols and sphingolipids influences insulin sensitivity and mitochondrial function in human skeletal muscle. JCI Insight. 2018;3(3):e96805. 29415895 10.1172/jci.insight.96805 PMC5821197

[75] Thiam AR , Beller M . The why, when and how of lipid droplet diversity. J Cell Sci. 2017;130(2):315‐324. 28049719 10.1242/jcs.192021

[76] Murphy DJ . The dynamic roles of intracellular lipid droplets: from archaea to mammals. Protoplasma. 2012;249(3):541‐585. 22002710 10.1007/s00709-011-0329-7

[77] Hesselink MK , Mensink M , Schrauwen P . Intramyocellular lipids and insulin sensitivity: does size really matter? Obes Res. 2004;12(5):741‐742. 15166292 10.1038/oby.2004.88

[78] Vock R , Hoppeler H , Claassen H , et al. Design of the oxygen and substrate pathways. VI. Structural basis of intracellular substrate supply to mitochondria in muscle cells. J Exp Biol. 1996;199(Pt 8):1689‐1697. 8708576 10.1242/jeb.199.8.1689

[79] Clerk LH , Vincent MA , Jahn LA , Liu Z , Lindner JR , Barrett EJ . Obesity blunts insulin‐mediated microvascular recruitment in human forearm muscle. Diabetes. 2006;55(5):1436‐1442. 16644702 10.2337/db05-1373

[80] McClatchey PM , Williams IM , Xu Z , et al. Perfusion controls muscle glucose uptake by altering the rate of glucose dispersion in vivo. Am J Physiol Endocrinol Metab. 2019;317(6):E1022‐E1036. 31526289 10.1152/ajpendo.00260.2019 PMC6957378

[81] Fazakerley DJ , Krycer JR , Kearney AL , Hocking SL , James DE . Muscle and adipose tissue insulin resistance: malady without mechanism? J Lipid Res. 2019;60(10):1720‐1732. 30054342 10.1194/jlr.R087510 PMC6795081

[82] Nuutila P , Knuuti MJ , Mäki M , et al. Gender and insulin sensitivity in the heart and in skeletal muscles. Studies using positron emission tomography. Diabetes. 1995;44(1):31‐36. 7813811 10.2337/diab.44.1.31

[83] Tramunt B , Smati S , Grandgeorge N , et al. Sex differences in metabolic regulation and diabetes susceptibility. Diabetologia. 2020;63(3):453‐461. 31754750 10.1007/s00125-019-05040-3 PMC6997275

[84] DeFronzo RA , Tobin JD , Andres R . Glucose clamp technique: a method for quantifying insulin secretion and resistance. Am J Phys. 1979;237(3):E214‐E223. 10.1152/ajpendo.1979.237.3.E214 382871

[85] Ascaso JF , Pardo S , Real JT , Lorente RI , Priego A , Carmena R . Diagnosing insulin resistance by simple quantitative Methods in subjects with normal glucose metabolism. Diabetes Care. 2003;26(12):3320‐3325. 14633821 10.2337/diacare.26.12.3320

[86] Reeder SB , Cruite I , Hamilton G , Sirlin CB . Quantitative assessment of liver fat with magnetic resonance imaging and spectroscopy. J Magn Reson Imaging. 2011;34(4):729‐749. 22025886 10.1002/jmri.22580 PMC3177109

[87] Ryan BJ , Schleh MW , Ahn C , et al. Moderate‐intensity exercise and high‐intensity interval training affect insulin sensitivity similarly in obese adults. J Clin Endocrinol Metab. 2020;105(8):e2941‐e2959. 32492705 10.1210/clinem/dgaa345 PMC7347288

[88] Newsom SA , Schenk S , Thomas KM , et al. Energy deficit after exercise augments lipid mobilization but does not contribute to the exercise‐induced increase in insulin sensitivity. J Appl Physiol. 2010;108(3):554‐560. 20044472 10.1152/japplphysiol.01106.2009 PMC2838634

[89] Steele R . Influences of glucose loading and of injected insulin on hepatic glucose output. Ann N Y Acad Sci. 1959;82:420‐430. 13833973 10.1111/j.1749-6632.1959.tb44923.x

